# TLN-4601 suppresses growth and induces apoptosis of pancreatic carcinoma cells through inhibition of Ras-ERK MAPK signaling

**DOI:** 10.1186/1750-2187-5-18

**Published:** 2010-11-02

**Authors:** Paul M Campbell, Nadia Boufaied, James J Fiordalisi, Adrienne D Cox, Pierre Falardeau, Channing J Der, Henriette Gourdeau

**Affiliations:** 1 Lineberger Comprehensive Cancer Center and Department of Pharmacology, University of North Carolina at Chapel Hill, Chapel Hill NC 27599-7295, USA; 2 Thallion PharmaceuticalsInc., 7150 Alexander-Fleming, Montreal QC, H4S 2C8 Canada; 3 Lineberger Comprehensive Cancer Center and Department of Radiation Oncology, University of North Carolina at Chapel Hill, Chapel Hill NC 27599-7295, USA

## Abstract

**Background:**

TLN-4601 is a structurally novel farnesylated dibenzodiazepinone discovered using Thallion's proprietary DECIPHER^® ^technology, a genomics and bioinformatics platform that predicts the chemical structures of secondary metabolites based on gene sequences obtained by scanning bacterial genomes. Our recent studies suggest that TLN-4601 inhibits the Ras-ERK MAPK pathway post Ras prenylation and prior to MEK activation. The Ras-ERK MAPK signaling pathway is a well-validated oncogenic cascade based on its central role in regulating the growth and survival of cells from a broad spectrum of human tumors. Furthermore, *RAS *isoforms are the most frequently mutated oncogenes, occurring in approximately 30% of all human cancers, and *KRAS *is the most commonly mutated *RAS *gene, with a greater than 90% incidence of mutation in pancreatic cancer.

**Results:**

To evaluate whether TLN-4601 interferes with K-Ras signaling, we utilized human pancreatic epithelial cells and demonstrate that TLN-4601 treatment resulted in a dose- and time-dependent inhibition of Ras-ERK MAPK signaling. The compound also reduced Ras-GTP levels and induced apoptosis. Finally, treatment of MIA PaCa-2 tumor-bearing mice with TLN-4601 resulted in antitumor activity and decreased tumor Raf-1 protein levels.

**Conclusion:**

These data, together with phase I/II clinical data showing tolerability of TLN-4601, support conducting a clinical trial in advanced pancreatic cancer patients.

## Background

Pancreatic ductal adenocarcinoma (PDAC) is the fourth leading cause of cancer death in North America and has a five-year survival rate of less than 5% [1]. Most patients with pancreatic cancer will die within six months of initial diagnosis. This poor prognosis has been related to the difficulty of detection in early stages of development, resulting in advanced disease at the time of presentation of first symptoms.

To acquire malignancy, pancreatic ductal epithelial cells undergo a series of sequential genetic mutations. Among the initial events are *KRAS *mutations and *HER-2*/*neu *amplification, followed by the loss of p16INK4A/CDKN2 expression and then inactivation of *p53 *and *DPC4*/*SMAD4 *[2-4]. *KRAS *mutations occur in almost all cases of pancreatic cancer. The most common alterations are substitutions at the codon 12 glycine, producing constitutively active K-Ras [4-6]. K-Ras is a small GTPase that is a key player in various signaling pathways, working as a molecular switch to transmit signals from the cell membrane to the cytoplasm and nucleus [7,8]. A variety of extracellular signals (hormones and growth factors) activate Ras by causing the exchange of GDP with GTP. In one of the canonical signaling pathways, K-Ras recruits Raf kinases (Raf-1, B-Raf, or A-Raf) to the cell membrane where their own activation takes place. Once activated, Raf phosphorylates mitogen-activated protein kinases (MEK1/2), which in turn phosphorylate and activate extracellular signal-regulated kinases (ERK1/2) [9].

Pancreatic cancer therapy has been a challenging issue. Since the approval of gemcitabine a decade ago following only modest increases in efficacy over 5-fluorouracil [10], none of the phase III clinical trials of newer cytotoxic or biologic agents combined with gemcitabine showed significant improvements in clinical benefit and survival (reviewed in [11]). A recent phase III trial combining erlotinib, an oral HER1/EGFR tyrosine kinase inhibitor, with gemcitabine is the first to demonstrate statistically significant improved survival in advanced pancreatic cancer [12]. This regimen is now considered to be a standard of care for pancreatic cancer, although the results of the erlotinib trial, while significant, resulted in only modest efficacy. The median survival on the combination was 6.24 months versus 5.91 months on gemcitabine alone, and the 1-year survival rates were 23% with the combination versus 17% with gemcitabine alone [12]. Therefore, while erlotinib in combination with gemcitabine is a step forward, there is still an urgent need to develop more effective drugs. Recently, a median survival of 11.1 months was achieved increasing the 1-year survival rate to 48% in a randomized phase III study on 342 patients receiving Folfirinox chemotherapy [13]. While gemcitabine has been the cornerstone for treatment of metastatic pancreatic adenocarcinoma, new and promising therapies are arising.

TLN-4601 (MW 462; US Patent 7,101,872) is a secondary metabolite produced by *Micromonospora *sp. This drug has been discovered through Thallion's DECIPHER^® ^platform, a genome scanning technique that allows prediction of the production and structure of secondary metabolites as well as facilitating their isolation [14-17]. Although the precise mechanism of action of TLN-4601 is unknown, our earlier work [18] indicated that TLN-4601 inhibits the EGF-induced Ras-ERK MAPK signaling pathway post Ras prenylation and prior to MEK activation and is mechanistically different from current Raf inhibitors and Ras-signaling inhibitors. The inhibition of growth factor-induced Ras-ERK MAPK signaling in human breast (MCF7) cells was not through direct kinase inhibition but rather through a proteasomal-dependent Raf-1 protein degradation [18]. Interestingly, these recent studies indicated that TLN-4601 does not bind to the conserved binding pocket domain of HSP90, suggesting a different mechanism of action to current HSP90 inhibitors. While it is common in breast cancer to see activation of Ras driven by aberrant upstream signaling through EGFR, VEGFR, etc. [19-22], the predominant cause of GTP-bound K-Ras in PDAC is a result of the aforementioned gene point mutations [4,6]. As such, it is important to further investigate TLN-4601 as a potential anti-tumorigenic agent in a relevant mutationally-activated K-Ras model. In the present study, we examined whether TLN-4601 could also inhibit oncogenic K-Ras-mediated signaling and tumorigenicity, which incorporates contributions from effector pathways in addition to Raf, by evaluating its effect on human PDAC and immortalized pancreatic ductal epithelial cells. Our results indicate that TLN-4601 decreases activated K-Ras (K-Ras-GTP) and Raf-1, which are associated with the inhibition of K-Ras-induced phosphorylation of MEK kinase. Moreover, we demonstrate that TLN-4601 induces apoptosis and inhibits the growth of human pancreatic (MIA PaCa-2) tumor xenografts.

## Results

### TLN-4601 inhibits contact-dependent and -independent growth in PDAC and immortalized pancreatic duct-derived cells

**Figure 1:**
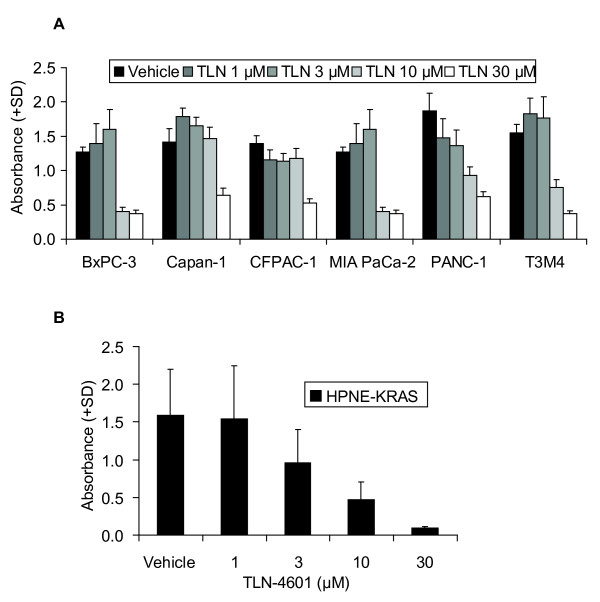
**TLN-4601 reduces contact-dependent proliferation in PDAC (A) and Ras-transformed immortalized duct derived cells (B)**. Cells were treated for 72 h with vehicle or various concentrations of TLN-4601 prior to the addition of the MTT reagent. Data represent the average of quadruplicate wells + SD of three independent experiments.

**Figure 2:**
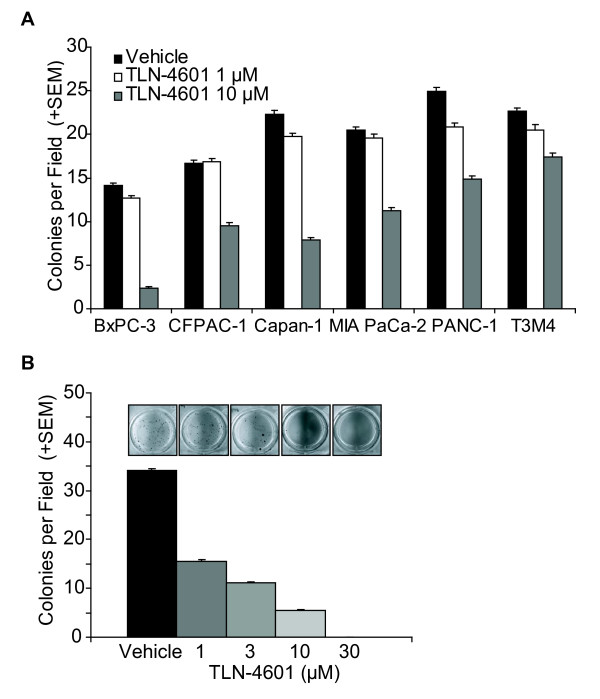
**TLN-4601 reduces contact-independent proliferation in PDAC (A) and Ras-transformed immortalized duct derived cells (B)**. Cells were seeded into soft agar containing vehicle or various concentrations of TLN-4601. Bars represent the mean colonies per field, averaged from five fields per well, triplicate wells + SEM, n = 3 independent experiments. Insets are representative wells.

To evaluate the effect of TLN-4601 on growth phenotypes shown to be increased in pancreatic cancer, we utilized both PDAC and genetically-engineered pancreatic cells expressing oncogenic K-Ras12D (HPNE-KRAS). Treatment with TLN-4601 resulted in a dose-dependent growth reduction of all six PDAC cell lines tested compared to vehicle controls (Figure 1A), regardless of their *KRAS *status. Similarly, TLN-4601 inhibited growth of immortalized human pancreatic duct-derived cells expressing mutant K-Ras12D protein (Figure 1B). These results indicate that TLN-4601-mediated inhibition of cell proliferation is not dependent on its ability to block oncogenic K-Ras function. We have previously demonstrated that several PDAC cell lines are capable of avoiding death upon release from a solid substrate [23], and have similarly demonstrated that expression of oncogenic K-Ras12D renders the same ability to immortalized pancreatic duct-derived cells [24,25]. Using the soft agar assay, our data indicate that TLN-4601 inhibits contact-independent colony formation of both pancreatic tumor cells and genetically-transformed pancreatic cells in a dose-dependent manner (Figure 2A, B). Thus, TLN-4601 inhibits at least one property of transformed cells that is dependent upon the activity of oncogenic K-Ras, ruling out a nonspecific toxic effect of this compound.

### TLN-4601 inhibits the oncogenic K-Ras-MAPK signaling pathway in PDAC cells

**Figure 3:**
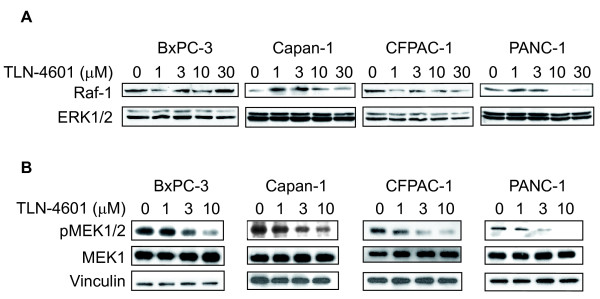
**TLN-4601 inhibits the K-Ras downstream signaling cascade in PDAC cells**. Exponentially growing cells were treated with increasing concentrations of TLN-4601. After overnight exposure, cells were lysed and cellular extracts (30 μg protein) were separated on a 10% SDS-PAGE gel and transferred to nitrocellulose membranes. (A) Membranes were sequentially probed with Raf-1 and ERK1/2 (p44/42 MAP kinase) or (B) phospho-MEK1/2 (Ser217/221), MEK1 and vinculin (loading control). Blots are representatives of three independent experiments.

We have previously illustrated that TLN-4601 inhibits the EGF-induced Ras-ERK MAPK signaling pathway in human breast tumor (MCF7) cells [18]. To evaluate the effect of TLN-4601 on the K-Ras signaling pathway in pancreatic cells, we used several PDAC cancer cell lines. Many PDAC cell lines have an activated Ras-ERK MAPK signaling pathway, indicated by the presence of phosphorylated MEK [26,27]. TLN-4601 modestly diminished Raf-1 protein levels in some but not all PDAC lines tested (Figure 3A). More striking, however, was the dose-dependent decrease in the activation/phosphorylation of MEK1/2 (pMEK1/2) in PDAC lines evaluated (Figure 3B). The protein levels of MEK1/2 and ERK1/2 were not affected by TLN-4601, suggesting that TLN-4601 acts upstream of both MEK and ERK. These results extend and confirm our previous findings [18].

**Figure 4:**
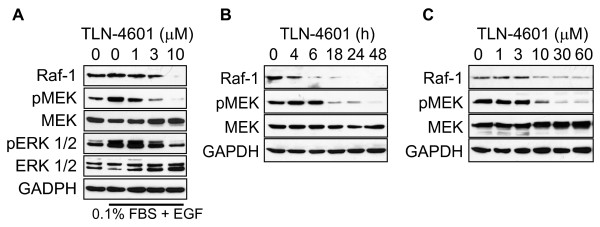
**TLN-4601 reduces K-Ras related signaling in EGF- (A) and serum-stimulated MIA PaCa-2 cells (B,C)**. Cells, treated with either escalating doses (A, C) or increasing time (B) of TLN-4601, were harvested and lysed for western blot analysis. GAPDH was used as a loading control.

We chose to focus on a single PDAC cell line (MIA PaCa-2) harboring a K-Ras mutation at codon 12 to further investigate the effects of TLN-4601 on the Ras-ERK MAPK signaling pathway. In cells either stimulated by EGF (Figure 4A) or growing in normal (10% serum) conditions (Figure 4B, C), overnight exposure to TLN-4601 resulted in a dose-dependent decrease in Raf-1 protein and pMEK1/2 (Figure 4A, C). These results confirm that TLN-4601 inhibits EGF-induced as well as constitutively activated Ras-ERK MAPK signaling in a rapid and persistent manner. A time course evaluation done at a fixed concentration of drug (10 μM) illustrates that these effects are observed as early as 4-6 h after treatment (Figure 4B), and that reduction of Raf-1 protein levels precedes the reduction in pMEK1/2. This suggests that TLN-4601 acts upstream of MEK1/2 activation.

### TLN-4601 inhibits Ras activation (Ras-GTP) in pancreatic cancer cells

**Figure 5:**
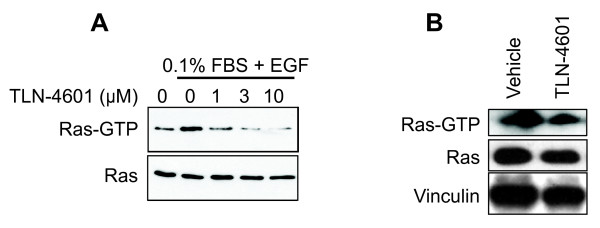
**Dose-dependent reduction of K-Ras GTP in MIA PaCa-2 (A) and immortalized K-Ras12D expressing cells (B).** Exponentially growing MIA PaCa-2 cells serum-starved starved for 9 h in DMEM with 0.1% FBS and then treated for 18 h with 0 (DMSO control), 1, 3 or 10 μM of TLN-4601. At the end of the treatment, cells were stimulated with EGF (100 mg/ml) for 5 min. HPNE-KRAS cells were grown in normal serum condition and treated for 18 h with 10 μM of TLN-4601. Pull-down analyses and western blot analyses with K-Ras-specific antibody were done to determine the level of activated and total K-Ras protein expression. Data are representative of one to three independent experiments. Vinculin was used as a loading control.

To discover whether the disruption of downstream Ras signaling in pancreatic cells might be in part due to a loss of Ras activation, a pull-down assay was used to study the effect of TLN-4601 on Ras-GTP in MIA PaCa-2 cells. This protocol takes advantage of the fact that the Ras binding domain (RBD) of Raf-1 binds preferentially to the activated form of Ras (Ras-GTP) [28-31]. Ras is already activated in the MIA PaCa-2 cell line and EGF stimulation had a minor effect on Ras-GTP levels. TLN-4601 treatment reduced EGF-induced Ras activation in a dose-dependent fashion (Figure 5A), while it did not affect total Ras protein expression (shown by western blotting of total cell lysates obtained prior to the pull-down fractionation). A reduction in Ras-GTP levels following TLN-4601 treatment was also observed in immortalized pancreatic cells expressing and dependent on mutant Ras (Figure 5B).

### TLN-4601 induces apoptosis in MIA PaCa-2 cells

**Figure 6:**
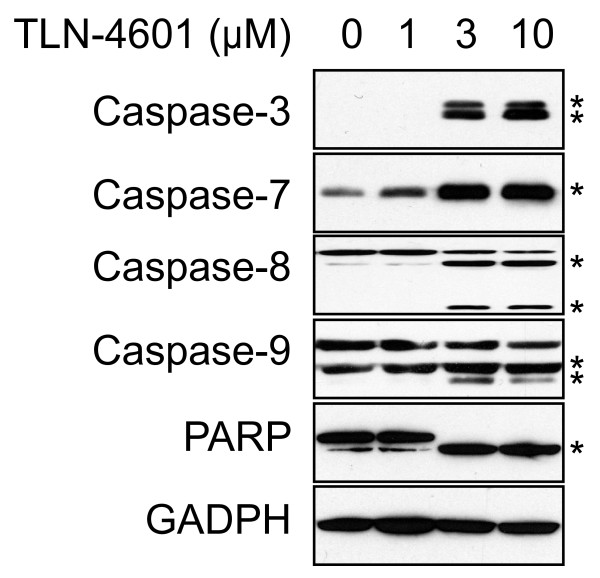
**TLN-4601 activates the apoptosis cascade in MIA PaCa-2 cells**. Lysate from TLN-4601-treated cells were separated by SDS-PAGE and probed for caspases and PARP activation and cleavage. GAPDH was used as a loading control. * Cleaved forms of different caspases and PARP

Activation of the Ras-ERK MAPK signaling pathway is essential for proliferation, differentiation and survival. Since TLN-4601 inhibits this pathway, we analyzed the effect of TLN-4601 on survival and apoptosis in MIA PaCa-2 cells exposed to increasing drug concentrations. As determined by MTT assay (Figure 1A), 10 μM TLN-4601 inhibited cell viability by more than 50%. TLN-4601 treatment resulted in activation of the initiator caspases 8 and 9 and the executor caspases 3 and 7 (Figure 6). Caspase activation leading to PARP cleavage was observed with 3 μM and 10 μM of TLN-4601, correlating with the MTT data. These results suggest that TLN-4601-driven diminishment of cell viability is at least in part due to the induction of apoptosis.

### TLN-4601 inhibits the growth of MIA PaCa-2 cell xenograft tumors

**Figure 7:**
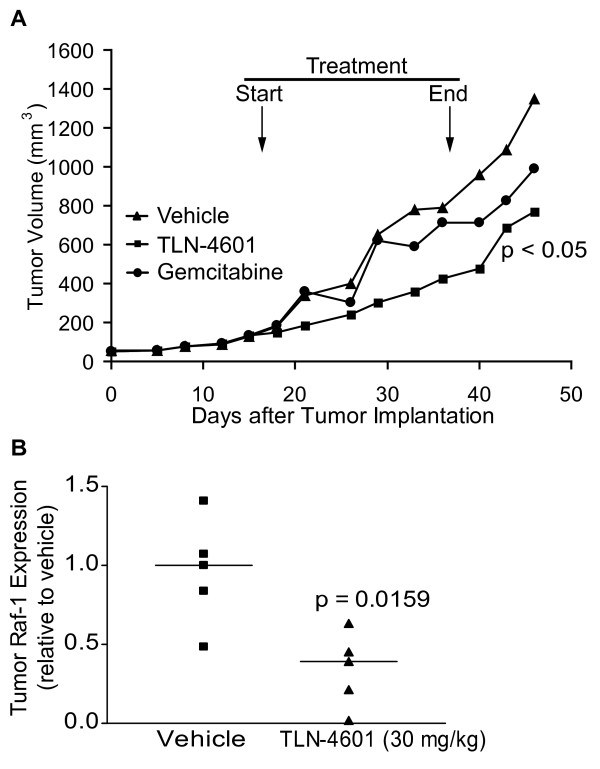
**TLN-4601 inhibits tumor growth and K-Ras signaling *in vivo***. MIA PaCa-2 xenograft-bearing nude mice were treated with TLN-4601 (30 mg/kg s.c., once a day Monday through Friday for three consecutive weeks) or gemcitabine (60 mg/kg i.p., twice a week for 4 weeks). Control group received 5 ml/kg of drug-free vehicle (15% PS80/5% PEG 400/5% EtOH/80% D5W) s.c., once a day Monday through Friday for three consecutive weeks. (A). Tumors were excised, lysed, and probed for Raf-1 protein expression (B); horizontal lines mark the mean of n = 5 mice.

After showing that TLN-4601 inhibits cell growth and transformation and induces apoptosis *in vitro*, we were interested in assessing its effect *in vivo*. Mice harboring MIA PaCa-2 tumor fragments were treated with 30 mg/kg of TLN-4601 daily from Monday to Friday for 3 consecutive weeks. Control mice were treated with either vehicle (negative control group) or gemcitabine (a standard treatment for pancreatic cancer). Treatment was started when the tumor xenograft had reached ~55 mm^3^, and the effect of compounds on tumor growth is shown in Figure 7A. Whereas the average tumor size increased from 57 to 1349 mm^3 ^in the control group (representing a 23.7 fold increase), average tumor size in the TLN-4601 treated group increased from 51 mm^3 ^to 769 mm^3^, representing a modest (T/C = 57%) but statistically significant reduction in tumor growth (43% tumor growth reduction; *p *= 0.009). While gemcitabine was administered at a dose and schedule reported to give optimal antitumor efficacy in some pancreatic tumor models [32], it did not result in significant antitumor efficacy in the MIA PaCa-2 xenograft model: the %T/C for that group, calculated at Day 49 (the time at which control mice were sacrificed due to tumor burden) was 73%, representing a 27% reduction in tumor growth (*p *= 0.73).

In order to correlate antitumor activity with the inhibition of the Ras-ERK MAPK signaling pathway by TLN-4601 observed *in vitro*, five additional mice bearing 100-200 mm^3 ^tumors were treated daily for 5 days with vehicle or 30 mg/kg TLN-4601. Tumor Raf-1 levels from TLN-4601-treated and control mice were compared by western blot analysis. As shown in Figure 7B, TLN-4601 treatment resulted in a decrease of Raf-1 protein levels in xenograft tumor tissues (*p *= 0.02), which is similar to the effects observed *in vitro*.

## Discussion

TLN-4601 is a structurally novel farnesylated dibenzodiazepine that has demonstrated antitumor efficacy against human hormone-independent breast and prostate tumor xenografts [33]. Its mechanism of action appears to be associated primarily with its inhibitory effect on Ras-ERK MAPK signaling [18]. The Ras-ERK MAPK signaling pathway regulates a large number of cellular processes, and a broad spectrum of human tumor types harbor mutations in this pathway leading to constitutive activation (reviewed in [34]). Pancreatic cancer is among these, with 90% of pancreatic cancers exhibiting *KRAS *mutations [35-37]. The importance of Ras in the initiation and progression of this disease has been previously reviewed [38]. Moreover, silencing mutant *KRAS *by RNA interference (RNAi) in Capan-2 human pancreatic cancer cells resulted in decreased *in vivo *tumorigenicity [39]. Taken together, these data indicate a critical role of K-Ras in pancreatic cancer and suggest that targeting mutant K-Ras specifically might be effective against pancreatic cancer *in vivo*.

We have previously reported that TLN-4601 inhibits cell proliferation and Ras-ERK signaling in EGF-stimulated human breast tumor cells with wild type K-Ras (MCF7) [18]. Here, we demonstrate that TLN-4601 suppresses proliferation, clonogenic survival and anchorage-independent growth of cell lines derived from malignant pancreatic tumors that harbor different oncogenic K-Ras mutations. Using the MIA PaCa-2 cell line as a model for pancreatic cancer, we showed that TLN-4601 resulted in a time- and concentration-dependent decrease in total Raf-1 protein levels that was associated with a subsequent inhibition of MEK phosphorylation. Together with our previous data showing that TLN-4601 inhibited Elk-1 transactivation by constitutively activated, N-terminally truncated Raf-1 BXB, but not by MEK [18], these observations indicate that TLN-4601 inhibits Ras-ERK signaling downstream or at the level of Raf-1 and upstream of MEK.

It is well documented that Raf is a major player in Ras-driven tumorigenesis. Engineered pancreatic duct-derived cells immortalized and rendered tumorigenic by ectopic expression of mutant K-Ras have shown that full K-Ras-dependent transformation requires Raf-MEK-ERK signaling [24]. The PI3K and the RalGDS pathways were also upregulated in these cells, but interestingly, inhibitors of the Raf-MEK-ERK cascade (U0126 and BAY 43-9006) were able to inhibit cellular transformation in this cell model system [24]. Increasing evidence indicates that Ras has several other effectors that contribute to Ras tumorigenic activities: phosphatidylinositol 3-kinases (PI3K), leading to AKT activation, RalGEF proteins and phospholipase Cε [40]. These Ras effectors also interact preferentially with GTP-bound Ras [41], but while it is clear that Raf-independent Ras-driven transformation contributes to tumorigenesis [42], the Ras-Raf-MEK-ERK cascade is still a very enticing area for cancer therapeutic development. In the present study, we demonstrate that TLN-4601 disrupts K-Ras signaling in transformed pancreatic cells by multiple events. First, TLN-4601 diminishes activated K-Ras-GTP levels, potentially blunting several oncogenic Ras effector pathways. In addition, TLN-4601 decreases cellular Raf-1 protein levels, inhibiting MEK phosphorylation and MAPK signaling. While the decrease in Raf-1 protein levels was not observed in all PDAC lines tested, TLN-4601 exposure was shown to result in a time- and dose-dependent decrease in MEK activation/phosphorylation in the PDAC lines evaluated. There are several effector pathways downstream of Ras, and the oncogenic or transformative contributions from the varied effectors differ from cell line to cell line and tumor to tumor. Given the complex regulation of MAPK activation and inactivation, both dependent on and independent of Ras (and Raf-1), not all cell lines depend solely upon Raf-1 for MAPK phosphorylation. Taken together, our data suggest that the drug may be acting by binding to and/or targeting a Ras scaffolding partner for degradation [43], or that TLN-4601 is capable of targeting Ras when the GTPase is in association with effector proteins such as Raf-1. Future experiments will focus on potential TLN-4601 intermediary targets.

Ras controls several aspect of malignant transformation, among them cell survival and apoptosis, where Ras activation suppresses apoptosis. In fact it has been shown that Ras-Raf-ERK and PI3K/AKT signaling pathways prevent apoptosis [44]. It is also well documented that Raf kinase plays an important pro-survival function that is completely independent of the MAPK signaling cascade [45]. Treatment with TLN-4601 decreases proliferation and induces apoptosis in MIA PaCa-2 cells, as shown by MTT assay, caspase activation, and PARP cleavage. By inhibiting the MAPK signaling pathway and inducing Raf-1 degradation, TLN-4601 may be sufficient to overcome Ras- and Raf-1-dependent pro-survival functions and drive cells into apoptosis. Interestingly, the PI3K-AKT signaling cascade appears unaffected by TLN-4601 treatment (data not shown), suggesting that specificity of action might somehow be conferred by a Ras-Raf-1-ERK complex that is independent of a PI3K-AKT axis. In this regard, it is important to note that the PI3K-AKT signaling pathway is commonly unlinked from Ras activation. Our previous analyses of pAKT levels in a large panel of *KRAS* mutant PDAC cells lines found infrequent AKT activation, suggesting that this effector pathway is not consistently activated by Ras activation in this tumor type [46]. This is also consistent with our published studies of Ras transformation of various epithelial cell types (RIE-1 intestinal and ROSE ovarian), where pAKT levels were not elevated by Ras activation [47,48].

We further extended our *in vitro *studies to an *in vivo *human pancreatic MIA PaCa-2 xenograft tumor model. Subcutaneous TLN-4601 administration resulted in moderate but statistically significant antitumor activity with a 43% overall reduction of tumor growth compared to the vehicle control group. Median tumor volume in the TLN-4601 cohort was significantly less than that of the gemcitabine treated group, 769 mm^3 ^and 991 mm^3^, respectively at Day 49, the time at which control mice were sacrificed due to tumor burden. The tumor growth inhibition was associated with a reduction in tumor Raf-1 protein levels. Raf-1 levels in tumors obtained from 5 mice treated with TLN-4601 were 50% of those found in tumors obtained from the vehicle-treated control group. The moderate antitumor activity can be explained by the rapid decay of circulating plasma TLN-4601 levels, as documented in a previous study [33]. Indeed, TLN-4601 is rapidly metabolized, and our PK/PD work led us to conclude that antitumor activity appears to be associated with the exposure parameter AUC and/or sustained drug levels rather than on short elevated systemic drug concentrations (Cmax). While TLN-4601 is given by continuous i.v. administration in cancer patients, this route of administration is not practical in mice. Preclinical antitumor evaluation is thus not performed at optimal drug concentrations.

In summary, TLN-4601 inhibits mutationally activated K-Ras-MAPK signaling and results in decreased *in vitro *contact-dependent and -independent growth of pancreatic cells, coupled with activation of apoptotic cascades. Furthermore, TLN-4601 demonstrated PDAC cell *in vivo *tumor xenograft growth inhibition, which was correlated with a reduction in tumor Raf-1 levels. These findings, together with phase I/II clinical data showing good safety and tolerability at drug plasma concentrations in the μM range [49], support further clinical development in mutated K-Ras-mediated cancers.

## Methods

### Cell culture and cell lines

PDAC cell lines, Capan-1 (mutated *KRAS*^G12V^), CFPAC-1 (mutated *KRAS*^G12V^), MIA PaCa-2 (mutated *KRAS*^G12C^), PANC-1 (mutated *KRAS*^G12D^), T3M4 (wild type *KRAS*), and BxPC-3 (wild type *KRAS*), were obtained from the American Type Culture Collection (ATCC; Manassas, VA). Cells were grown in Dulbecco's modified Eagle (DMEM) medium supplemented with 10% heat-inactivated fetal bovine serum (FBS; WISENT Inc., QC, Canada) and maintained in a humidified atmosphere at 37°C with 5% CO_2_. Cell lines were started from frozen stocks, maintained in culture for 15 to 20 passages and were free of *Mycoplasma *(routinely tested by PCR; Sigma-Aldrich).

Normal human immortalized pancreatic duct-derived cells (HPNE) with mutant K-Ras^G12D ^expression (HPNE-KRAS) were obtained as previously reported [24,50]. These cells were maintained at 5% CO_2 _in M3:5 growth medium [4 parts high-glucose DMEM (Life Technologies, Carlsbad, CA) to 1 part M3F (INCELL, San Antonio, TX) supplemented with 5% FBS.

### Cell viability assays

Exponentially growing cells were plated into 96-well plates (5 × 10^3 ^in 150 μl/well), and 18 h later treated with increasing concentrations of TLN-4601 for 72 h. At the end of the treatment, 20 μl of 5 mg/ml 3-(4,5-dimethylthiazol-2-yl)-2,5-diphenyltetrazolium bromide (MTT, Sigma-Aldrich) was added to each well and the plates were incubated for an additional 4 h at 37°C. Following this, the medium was removed and replaced with 200 μl of dimethylsulfoxide (DMSO). Experiments were done in quadruplicate and repeated two to three times. The absorbance at 570 nm was measured by plate reader.

Contact-independent growth analysis was performed according to previous protocols [24]. Briefly, log phase growing cells were trypsinized, and triplicates of 3 × 10^3 ^cells per well were suspended in enriched medium (supplemented with an additional 10% fetal calf serum) mixed with 1.5% sterile agar and plated onto dense agar coated six-well plates. One ml of standard medium was added to the top of the gelled matrix and colonies were grown for 21 days. Stock solutions of TLN-4601 were dissolved in DMSO and added to both the agar containing the cells and the feeding medium. After 21 days in culture, live colonies were counted in five random three-dimensional fields per well, stained with MTT, and photographed.

### Cell lysis and western blots

After treatment, cells were washed twice with PBS, lysed in lysis buffer [50 mM Tris-HCl pH 7.4, 1% Triton X-100, 1% sodium deoxycholate, 0.1% SDS, 150 mM NaCl, 1 mM EDTA, phosphatase inhibitor cocktail set II (EMD Biosciences, Calbiochem, San Diego, CA) and protease inhibitor cocktail (Roche, Mannheim, Germany)] and cleared by centrifugation at 14000 × *g *for 10 min. Total proteins (30 μg) were separated by SDS-PAGE, transferred onto nitrocellulose or PVDF membranes, blocked in 5% nonfat dry milk in TBST and probed with anti- Raf-1, anti- MEK1/2, anti-ERK1/2, anti-PARP (all from Cell Signaling Technology, Beverly MA), anti-phospho-MEK1/2, anti-phospho-ERK1/2, anti-vinculin and anti-GAPDH (all from Santa Cruz Biotechnology, Santa Monica, CA). Primary antibodies were detected with horseradish peroxidase-conjugated secondary antibodies and chemiluminescent HRP substrate (Millipore, Mississauga, ON, Canada).

### GTP pull-down assays

MIA PaCa-2 cells were cultured in DMEM medium supplemented with 10% FBS for 18 h, starved for 9 h in DMEM 0.1% FBS and then treated for 18 h with increasing concentrations of TLN-4601. At the end of the treatment, cells were stimulated with EGF (100 mg/ml) for 5 min. HPNE-KRAS cells were grown in M3:5 growth medium supplemented with 5% FBS and treated for 18 hours with increasing concentrations of TLN-4601. Ras GTP levels were determined by a Ras activation assay kit (Upstate, Millipore) according to the manufacturer's directions, or by previously published protocols [24,51]. Lysates (1 mg of total cell protein in each sample) were incubated with 10 μg Raf-1-RBD for 45 min at 4°C and centrifuged for 15 sec at 14000 × *g *to pellet the agarose beads. After discarding the supernatant, agarose beads were washed three times with 500 μl of lysis buffer and the pellets were resuspended in 2× Laemmli sample buffer containing DTT, boiled for 5 min, and centrifuged at 14000 × *g*. The supernatant was collected and cellular proteins resolved by 12% SDS-PAGE and analyzed by western blotting using a K-Ras specific antibody.

### Animal studies

The xenograft MIA PaCa-2 pancreatic carcinoma donor tumor was generated by injecting 2 × 10^7 ^MIA PaCa-2 cells into the right flanks of female Swiss nude mice (total of 10 mice). Those tumors were excised and small fragments (~1 mm^3^) were implanted s.c. into the right flanks of female Swiss nude mice (8-9 weeks old). When tumor volumes reached 50-60 mm^3^, mice were randomized into groups of 10-15 mice and treated. The study involved a negative control group (vehicle-treated), a gemcitabine-treated group (60 mg/kg i.p., once a day, twice per week for four weeks), and a TLN-4601 treated group (30 mg/kg s.c., once a day Monday through Friday for 3 consecutive weeks). The study was performed at OncoDesign, Dijon, France in accordance with the recommendations of the French Ethics Committee and under the supervision of authorized investigators. Tumor growth was followed twice a week by measuring tumor length (L) and width (W) using a caliper. Measurements were converted to tumor volumes (TV; mm^3^) using the standard formula, TV = (L × W)^2^/2. Animals were sacrificed when tumors in the control group reached a predetermined endpoint TV of ~1400 mm^3 ^(Day 49). Compound efficacy was assessed by percentage of treated vs control (%T/C) defined as the (median treated tumor volume/median control tumor volume × 100). Tumor growth reduction was calculated by subtracting the % T/C from 100.

Evaluation of Raf-1 tumor levels was performed in a subset of five mice each from the control and TLN-4601 treated groups after the first five days of treatment. Tumor lysates were prepared by sonication in lysis buffer and samples were processed as described above.

Statistical analysis was done by ANOVA or Student's *t *test. Differences were considered to be significant at p < 0.05.

## List of abbreviations

DMEM: Dulbecco's modified Eagle medium; DMSO: dimethylsulfoxide; EGF: extracellular growth factor; DTT: dithiothreitol; EGFR: extracellular growth factor receptor; ERK: extracellular signal-regulated kinase; GDP: guanosine diphosphate; GEF: guanine nucleotide exchange factor; GTP: guanosine triphosphate; GTPase: guanosine triphosphatase; HPNE: human pancreatic duct-derived nestin expressing cell; i.p.: intraperitoneal; i.v.: intravenous; MAPK/MEK: mitogen-activated protein kinase; MTT: 3-(4,5-dimethylthiazol-2-yl)-2,5-diphenyltetrazolium bromide; PAGE: polyacrylamide gel electrophoresis: PARP: poly (ADP-ribose) polymerase; PDAC: pancreatic ductal adenocarcinoma; PBS: phosphate buffered saline; PI3K: phosphatidylinositol 3-kinase; PVDF: polyvinylidene fluoride; RalGDS: Ral guanine nucleotide dissociation stimulator; RBD: Ras binding domain; s.c.: subcutaneous; SDS: sodium dodecyl sulfate; VEGFR: vascular endothelial growth factor receptor.

## Competing interests

NB, PF and HG were employees of Thallion Pharmaceuticals at the time of the study and PF and HG own stock options. The work presented here was funded by Thallion Pharmaceuticals Inc. and a PCT application (WO2009-124399A1) has been filed.

## Authors' contributions

PMC performed cell viability and clonogenic assays, immunoblotting, oncogene activation assays and drafted the manuscript. NB carried out the apoptosis analysis, immunoblotting, oncogene activation assays, and contributed to the drafting of the manuscript. JJF carried out immunoassays. ADC, PF, CJD, and HG all participated in the design and coordination of the study and helped to draft the manuscript. All authors read and approved the final manuscript.
